# Evaluation of the association of endometriosis and mammographic breast density, a cross-sectional study

**DOI:** 10.1186/s12905-022-01663-8

**Published:** 2022-03-21

**Authors:** Ashraf Moini, Elnaz Salari, Hadi Rashidi, Khadije Maajani, Mahboubeh Abedi, Leila Bayani, Sadaf Alipour

**Affiliations:** 1 Breast Disease Research Center (BDRC), Tehran University of Medical Sciences, Tehran, Iran; 2 Department of Surgery, Arash Women’s Hospital, Tehran University of Medical Sciences, Shahid Baghdarnia (North Rashid) Street, Ressalat Street, 1653915911 Tehran, Iran; 3 Department of Endocrinology and Female Infertility, Reproductive Biomedicine Research Center, Royan Institute for Reproductive Biomedicine, ACECR, Tehran, Iran; 4 Department of Epidemiology and Biostatistics, School of Public Health, Tehran University of Medical Sciences, Tehran, Iran; 5 Department of Radiology, Arash Women’s Hospital, Tehran University of Medical Sciences, Tehran, Iran; 6 Department of Surgery, Arash Women’s Hospital, Tehran University of Medical Sciences, Tehran, Iran

**Keywords:** Endometriosis, Mammographic density, Breast cancer, Women, Iran

## Abstract

**Background:**

Endometriosis is a common benign but painful gynecologic condition. Studies suggest that the risk of some types of malignancies such as breast cancer is higher in women with endometriosis. Mammographic breast density (MBD) is known as an important predictor for breast cancer. The present study aimed to investigate the potential relationship between endometriosis and MBD.

**Methods:**

This cross-sectional study was conducted on 370 women over 40 years of age. Laparoscopic surgery was carried out for the diagnosis of endometriosis. MBD was classified into four categories according to the ACR BI-RADS classification. Statistical analysis was performed using SPSS software to evaluate the potential association between variables.

**Results:**

The mean age of all participants was 47.2 ± 6.4 years, and most participants (76.8%) were premenopausal. Multivariate analysis of the potential predictors of MBD, including age, body mass index, oral contraceptive consumption, progesterone consumption, family history of breast cancer and endometriosis showed that age (*P* value = 0.002), history of progesterone consumption (*P* value = 0.004) and endometriosis (*P* value = 0.006) were independent factors for MBD.

**Conclusion:**

This study indicated that endometriosis had an inverse association with MBD. Age and history of progesterone use were also independent influential factors for MBD. This finding shows that the positive association between breast cancer and endometriosis is not mediated through MBD.

## Introduction

Endometriosis is a painful gynecologic condition defined by the presence of endometrial-like tissue outside the uterus [1]. As one of the most prevalent benign disorders of the female genital system, endometriosis is a debilitating disease with detrimental effects on social, occupational and psychological functioning [2]. There are some similarities between endometriosis and female malignancies: progressive and invasive growth, estrogen-dependency, recurrence and tendency to metastasize [3]. According to the different epidemiological studies around the world, endometriosis affects about 10% of women at reproductive age and 30–50% of those who are suffering from chronic pelvic pain or infertility, which are the two major clinical symptoms of endometriosis [4]. Several possible etiopathogenic mechanisms have been put forward for endometriosis. Among them, mutations in several genes and polymorphism are being strongly suggested as risk factors for endometriosis, these can be inherited in families [5, 6]. Another interesting theory regarding the etiology of endometriosis consists of the effect of metabolic alterations, which has been assessed by metabolomics in studies [7]. Also, increases in the permeability of the small intestine and translocation of lipopolysaccharides from the bowels to the peritoneal cavity have been mentioned as causes for the chronic inflammation in endometriosis [8]. Although none of these have been studied as s a factor affecting MBD, but one more recognized etiology of endometriosis can affect MBD: it is known that sex steroid hormones have a key role in endometriosis development and progression [1]. Existing evidence suggests that the risk of some chronic diseases like cardiovascular disease, and some types of malignancies including ovarian and breast cancer might be higher in women with endometriosis [9]

Mammographic breast density (MBD), which indicates the fibro-glandular tissue content of the breast, is considered one of the important predictors for breast cancer among females in the general population. It has been shown that a high MBD (75% density) increases the risk of breast cancer by four-to-six folds in comparison to a low MBD (< 5% density) [10]. It is assumed that exposure to sex-steroid hormones may have a role in MBD, particularly, menopausal hormone replacement therapy increases MBD, while menopausal status and tamoxifen decrease it [11].

As sex steroid exposure is associated with both endometriosis and MBD, and both are related with breast cancer, we aimed to investigate the potential relationship between endometriosis and MBD in women over 40 years of age.

## Methods

This is a cross-sectional study carried out in Arash women’s hospital, Tehran, Iran. The study was approved by the Ethics Committee of Tehran University of Medical Sciences, Tehran, Iran (Approval ID: IR.TUMS.MEDICINE.REC.1398.130), and as a resident’s thesis by the Institutional Research Board of the University (Proposal Code: 961129000). All the protocols involving humans were in accordance to the Institutional Guidelines of Ethical Research of Tehran University of Medical Sciences and to the Declaration of Helsinki. Written informed consent was obtained from all participants.

The study was conducted on women over 40 years of age. According to a prevalence of 40% for high MBD reported in the study of Alipour et al. [12], and to detect a 15% difference in the prevalence of high MBD between the two groups, the estimated sample size was 180 for each group based on a power of 80% and a level of type I error of 5%. The final sample size was 360 plus 10 extra cases in the control group.

Women who were diagnosed with endometriosis by laparoscopy were considered as cases, and controls were selected from women who had previously undergone laparoscopic surgery due to any reason (pelvic pain, dysmenorrhea, unknown infertility, etc.), and in whom the absence of endometriosis was confirmed during the surgery. Exclusion criteria consisted of a history of any type of cancer or previous radiotherapy, a positive genetic test for breast cancer (BRCA1, BRCA2), a positive family history of breast or ovarian cancer in first degree relatives, or having had a mammogram less than 1 year sooner. The rational for choosing these criteria for excluding participants was that in our opinion, they could strongly affect the MBD independent of the endometriosis status of participants. For the last criterion, our study was prospective and the participant had to undergo mammography, so we could not enter women who had recently undergone this imaging.

Data regarding demographic information and other risk factors including reproductive features were obtained through interview. Then, all eligible participants underwent mammography in our radiology center. MBD was classified into four categories and defined according to the American College of Radiology (ACR) Breast Imaging Reporting and Data System (BI-RADS) by two expert radiologists [13]. Data was analyzed using SPSS software Version 26.

The continuous variables are reported as means ± SD, and numbers and percentages are used for reporting categorical variables. Normality for continuous variables was determined by the Kolmogorov–Smirnov test, which revealed the normal distribution of continuous variables (*P* > 0.05). The Independent T-Test, Pearson’s Chi-square and Fisher exact test were used for the comparison of differences between the variables in the study groups. Univariate and Multiple linear regression were applied to evaluate the possible association between endometriosis and potential risk factors. *P* values of < 0.05 were accepted as significant.

## Results

**Fig. 1:**
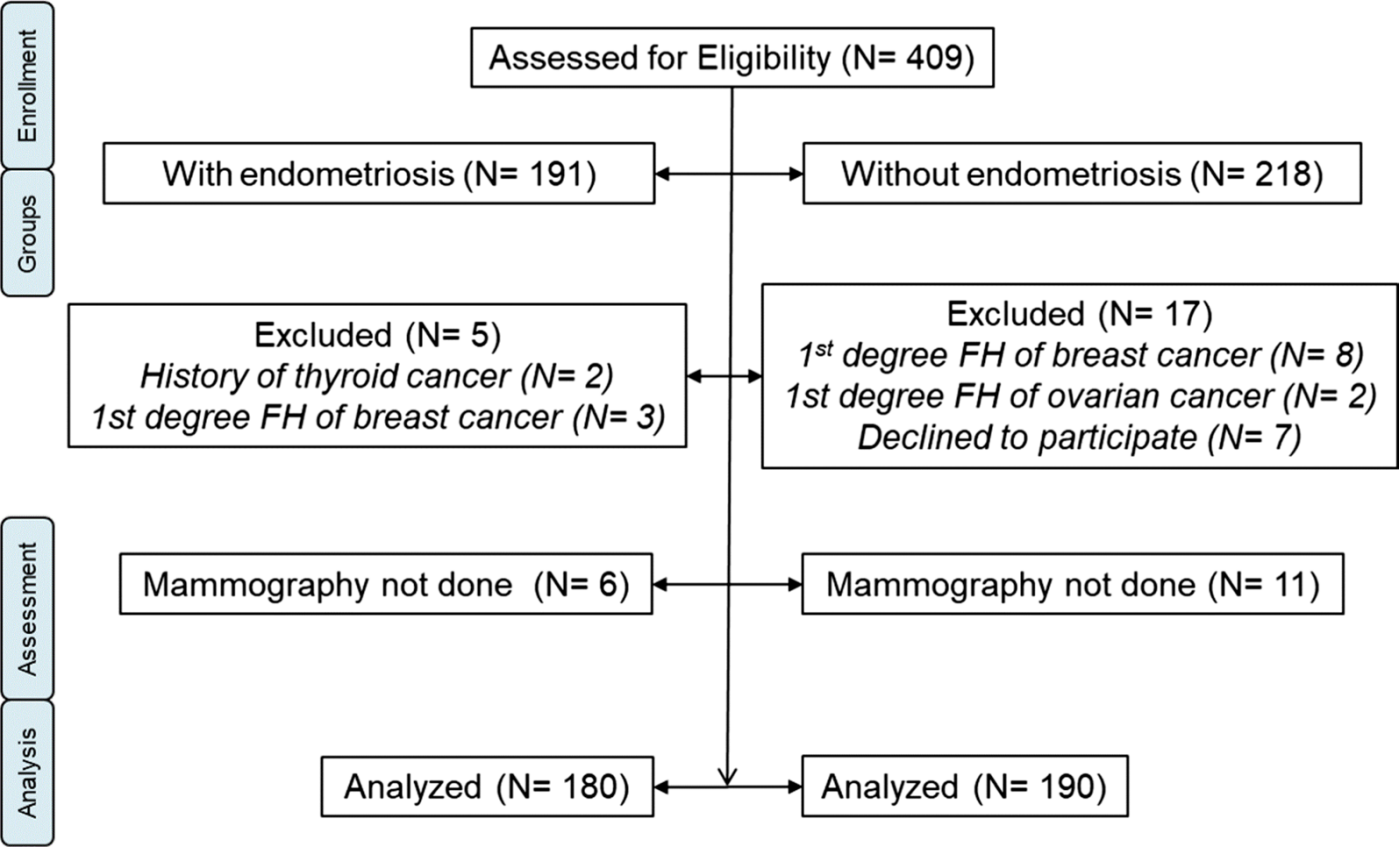
Flowchart of number of participants in the two groups. *FH* family history

The number of women assessed for eligibility, excluded cases and controls, those who were withdrawn and the total number analyzed are demonstrated in Fig. 1. A total of 370 women were entered into the study. The mean age of all participants was 47.2 ± 6.4; the youngest and oldest were 40 and 71 years old, respectively. Among all participants, 284 (76.8%) were premenopausal and 86 (23.2%) women were postmenopausal.

According to the analysis of demographic and clinical characteristics of participants, most of the variables were significantly different between the two groups, except for age at menarche, age at first pregnancy, duration of progesterone usage, history of infertility treatment, abortion, abdominal surgery and breast disease, which were not different between the two groups. The results are shown in Table 1.

**Table1:** Demographic and clinical characteristics in the two study groups

Characteristic	Cases (N = 180)	Controls (N = 190)	*P* value
Age	44.51 ± 4.40	49.85 ± 6.99	0.001
Parity	1.61 ± 1.22	2.47 ± 1.37	< 0.001
Gravidity	1.95 ± 1.40	2.8 ± 1.49	< 0.001
BMI	27 ± 4.27	28.7 ± 4.36	0.001
Age at menarche	13.41 ± 1.5	13.33 ± 1.20	0.57
Age at first pregnancy	22.45 ± 4.94	21.44 ± 5.12	0.07
Menopause age	46.15 ± 4	49.34 ± 4.82	0.002
OCP usage duration (year)	1.85 ± 2.43	3.51 ± 4.87	0.002
Progesterone usage duration (year)	1.41 ± 2.46	1.50 ± 2.45	0.86
Lactation duration			0.001
Never	42 (23.3%)	13(6.8%)	
Less than 6 months	3 (1.7%)	3(1.6%)	
7–12 month	0 (0%)	9 (4.7%)	
13–24 month	63 (35%)	73 (38.4%)	
More than 24 month	72 (40%)	92 (48.4%)	
Menopausal status	28 (15.4%)	58 (30.5%)	0.001
Infertility	49 (27.2%)	17 (8.9%)	0.001
Infertility treatment (n = 66)	33 (67%)	9 (52.9%)	0.28
History of miscarriage	50(27.8%)	59 (31.1%)	0.49
History of curettage	20(11.1%)	31 (16.3%)	0.14
OCP usage	114 (63.3%)	101 (53.2%)	0.047
Progesterone usage	93 (51.7%)	34 (17.9%)	0.001
Adenomyosis	27 (15%)	14 (7.4%)	0.01
Abdominal surgery	116 (64%)	108 (56.8%)	0.13
Dysmenorrhea	121 (67.2%)	92(48.4%)	0.001
Dyspareunia	78 (43.3%)	49 (25.8%)	0.001
Pelvic pain	111 (61.7%)	60 (31.6%)	0.001
Breast disease	49 (27.2%)	74 (38.9%)	0.01
Type of breast disease			0.32
Fibro adenoma (n = 48)	1 (2.1%)	4 (5.8%)	
Fibrocystic disease (n = 69)	47 (97.9%)	65(94.2%)	
2nd or 3rd degree family history of breast cancer	16 (8.9%)	33(17.4%)	0.01
Oophorectomy			0.01
Unilateral	4 (2.2%)	1 (0.5%)	
Bilateral	10 (5.6%)	0 (0%)	
Hysterectomy	16 (8.9)	4 (2.1)	0.004
Breast density			0.001
Grade1	113 (62.8%)	77 (40.5%)	
Grade2	56 (31.1%)	96 (50.5%)	
Grade3	11 (6.1%)	13 (6.8%)	
Grade4	0 (0%)	4 (2.1%)	

Univariate and multivariate analysis were carried out to understand the relative importance of potential predictors of MBD. Variables including age, body mass index (BMI), oral contraceptive (OCP) use, progesterone use, 2nd and 3rd degree family history of breast cancer and a family history of endometriosis were included as independent predictors for MBD. Univariate analysis revealed that endometriosis (*P* value = 0.001), as well as age (*P* value = 0.001) were associated with MBD. Consequently, the potential factors were included in multivariate analysis, and the results showed that endometriosis (*P* value = 0.006), age (*P* value = 0.002), and history of progesterone consumption (*P* value = 0.004) were independent factors for MBD (Table 2). According to results, MBD was higher in older women, and women with lower grade of MBD had a lower mean age (were younger). Regarding BMI, women with a grade 4 MBD had a significantly higher BMI (Table 3).

**Table 2:** Effect of predictor variable on breast density (as a continuous variable) using univariate and multiple linear regression

Variable	Univariate linear regression			Multivariate linear regression		
	β	SE	*P* value	β	SE	*P* value
Age	0.02	0.005	0.001	0.01	0.006	**0.002**
BMI	0.008	0.008	0.28	− 0.001	0.008	**0.88**
OCP (no, yes)	− 0.11	0.07	0.10	− 0.06	0.06	**0.35**
Progesterone (no, yes)	0.027	0.07	0.71	0.15	0.07	**0.04**
Family history of breast cancer (2nd and 3rd degree)	0.13	0.10	0.17	0.10	0.1	**0.31**
Endometriosis	− 0.27	0.06	0.001	− 0.21	0.07	**0.006**

The* p*-values are in bold

**Table 3:** Effect of suspected variables on different grades of ammographic breast density (as a categorical variable)

Mammographic breast density	Grade1	Grade2	Grade3	Grade4	*P* value
Age (Mean ± SD)	45.85 ± 5.76	48.44 ± 6.71	50.33 ± 7.69	50.25 ± 3.86	0.0001^a^
BMI (Mean ± SD)	27.83 ± 4.32	27.97 ± 4.45	27.51 ± 3.32	34.17 ± 6.73	0.038^a^
OCP (N, %)					
Yes	117 (54.4)	85 (39.5)	11 (5.1)	2 (0.9)	0.39^b^
No	73 (47.1)	67 (43.2)	13 (8.4)	2 (1.3)	
Progesterone (N, %)					
Yes	64 (50.4)	52 (40.9)	10 (7.9)	1 (0.8)	0.88^b^
No	126 (51.9)	100 (41.2)	14 (5.8)	3 (1.2)	
Family history of breast cancer (2nd and 3rd degree) (N, %)					
Yes	22 (44.9)	21 (42.9)	5 (10.2)	1 (2%)	0.35^b^
No	168 (52.3)	131(40.8)	19 (5.9)	3 (0.9)	
Endometriosis (N, %)					
Yes	113 (62.8)	56 (31.1)	11 (6.1)	0	0.0001^b^
No	77 (40.5)	96 (50.5)	13 (6.8)	4 (2.1)	

^a^One-way analysis of variance (ANOVA)

^b^Fisher exact test

## Discussion

In this study, we evaluated the association between endometriosis and MBD, and the risk factors of endometriosis in the case and control groups. We found that women with endometriosis had a lower MBD than those without endometriosis. Age and progesterone usage were the other predictors of MBD.

According to the studies around the world, the rate of diagnosing endometriosis is rising due to the increased awareness of women about the disease, changing social patterns like late marriage, and the widespread use of laparoscopy [14].

On the other hand, MBD is a potential risk factor for breast cancer. There are several studies that confirm the association between this cancer and MBD [15–18]. The risk of breast cancer according to MBD category varies by studies, a study reported that women with high MBD have two times a higher risk for this cancer [15]. Another study reported a four-to-six-fold risk of breast cancer in women with high MBD [19]. What stands out from these reports is that MBD has a major impact on breast malignancy. Thus, investigating the influential factors on MBD can play a major role in prevention and control of breast cancer, also evaluation of a possible association between endometriosis and MBD may pave the way to revealing the pathway from endometriosis to breast cancer.

According to our findings, endometriosis is a significant predictor for MBD; however in contrast with our expectation, MBD was lower in women with endometriosis. The mechanism for this reverse association is not clear to us, but this shows that the association of endometriosis and breast cancer is not through MBD. It also infers that sex hormones alone are not implicated in female cancers after endometriosis. To the best of our knowledge, the only study which evaluated the relationship between endometriosis and MBD was that of Farland et al. [20]. According to this study, endometriosis was not found to be associated with mammographic density, which was in contrast with our finding. However, our sample size was higher, and Farland et al. did not consider the use of steroid hormones as a confounding factor.

Age and progesterone use were the other variables that showed significant relationship with MBD. We found that a history of progesterone consumption was associated with a higher MBD. There are studies that are in agreement with our finding about the role of progesterone in MBD [21–24]. Those studies also reported that higher levels of progesterone were associated with greater MBD. This finding is not unexpected, as progesterone has a key role in regulation of tissue development and maturation in the young breast, and atrophy and involution of the lobules and ducts during and after menopause [25].

Among variables that were evaluated as influential factors for MBD, BMI and OCP usage were not significantly associated with MBD. These variables have been reported to be associated with MBD in some studies. For instance, in two different studies conducted by Alipour et al. [26] and Yang et al. [27], BMI was negatively correlated with MBD. In a study conducted on Chinese women, Shang et al. identified BMI as an independent influential factor on MBD [28].

Interestingly, several factors that were presumed as influencing both endometriosis and MBD have been studied previously. Low levels of serum Vitamin D have been assumed as a risk factor for endometriosis and for increased MBD. However, this relation has been highly demonstrated for the former [29], but not in the latter condition [30]. Also, use of dairy products might lower the risk of endometriosis [31], while the few studies about the association of these products with MBD have showed controversial results from no relation to a negative association [32].

Our study had some limitations. We did not classify endometriosis according to their severity and therefore we could not asses the association of different severity classes with MBD. Also, we did not include the duration of use of OCP and progesterone in our data, while this could have affected the results.

In conclusion, our study showed that endometriosis was inversely associated with BMD. Considering the increased risk of breast cancer in women with higher BMD, our findings show that were there a positive association between endometriosis and breast cancer, this is not mediated via MBD. Further studies are warranted to define the complex relations among endometriosis, MBD and breast cancer.

## Data Availability

Data are available per request from Elnaz Salari at dr.elnaz.salari@gmail.com.
